# In search of an ideal drug for safer treatment of obesity: The false promise of pseudoephedrine

**DOI:** 10.1007/s11154-021-09658-w

**Published:** 2021-05-04

**Authors:** Antonio Munafò, Stefano Frara, Norberto Perico, Rosaria Di Mauro, Monica Cortinovis, Chiara Burgaletto, Giuseppina Cantarella, Giuseppe Remuzzi, Andrea Giustina, Renato Bernardini

**Affiliations:** 1 Department of Biomedical and Biotechnological Sciences, University of Catania School of Medicine, Catania, Italy; 2 Institute of Endocrine and Metabolic Sciences (IEMS), San Raffaele Vita-Salute University, Milano, Milano Italy; 3 Istituto Di Ricerche Farmacologiche “Mario Negri”, Bergamo, Italy

**Keywords:** Dietary restriction, Obesity, Sympaticomimetic drugs, Severe adverse events

## Abstract

Obesity is a major public health problem worldwide. Only relatively few treatment options are, at present, available for the management of obese patients. Furthermore, treatment of obesity is affected by the widespread misuse of drugs and food supplements. *Ephedra sinica* is an old medicinal herb, commonly used in the treatment of respiratory tract diseases. *Ephedra* species contain several alkaloids, including pseudoephedrine, notably endowed with indirect sympathomimetic pharmacodynamic properties. The anorexigenic effect of pseudoephedrine is attributable primarily to the inhibition of neurons located in the hypothalamic paraventricular nucleus (PVN), mediating satiety stimuli. Pseudoephedrine influences lipolysis and thermogenesis through interaction with β3 adrenergic receptors and reduces fat accumulation through down-regulation of transcription factors related to lipogenesis. However, its use is associated with adverse events that involve to a large extent the cardiovascular and the central nervous system. Adverse events of pseudoephedrine also affect the eye, the intestine, and the skin, and, of relevance, sudden cardiovascular death related to dietary supplements containing *Ephedra* alkaloids has also been reported. In light of the limited availability of clinical data on pseudoephedrine in obesity, along with its significantly unbalanced risk/benefit profile, as well as of the psychophysical susceptibility of obese patients, it appears reasonable to preclude the prescription of pseudoephedrine in obese patients of any order and degree.

## Introduction

Obesity is a chronic and multifactorial [1] disease characterized by increased body weight due to an excessive fat accumulation as a result of daily intake excess and inadequate calorie expenditure [2]. Such imbalance determines, in the long run, an excess in adipose tissue that leads, firstly, to an overweight body phenotype and, at a later stage, to the development of a body weight disorder called obesity [3].

In previous years, different approaches to obesity have led to the development of new techniques to overcome the biases related to the obesity definition. Evaluation of the body composition by Dual Energy X-Ray Absorptiometry (DEXA) or electrical bio-impedance analysis (BIA) provided reliable data in many trials [4], but higher costs, radiation exposure, patient inconvenience and less availability represent limitations to the widespread of these technologies.

Obesity represents a worldwide health problem in adults, as well as among children and adolescents, and significantly increases the risk of developing metabolic syndrome, type 2 diabetes mellitus, hypertension and cardiovascular and kidney diseases leading to high all-cause mortality [5, 6]. In addition, numerous cohort studies have shown the link between obesity and the increased incidence of different types of cancer, including colon, postmenopausal breast, endometrial cancers and esophageal adenocarcinoma [7, 8].

Personalized dietary regimens [9] and physical activity are the cornerstones of anti-obesity therapy, which should be performed under medical supervision; however, this strategy is not easy to achieve as many patients show poor adherence and a low success rate [10]. Pharmacological therapy of obesity is considered a controversial issue because in many cases medicines have modest efficacy while exhibiting considerable adverse events [11]. The major pathways implicated in controlling metabolism and nutrient intake include the hypothalamic system leptin-melanocortin [12, 13], the adrenergic [14], cannabinoid [15], dopaminergic [16], and opioidergic [17] systems in the hypothalamus and other brain regions. These selected central nervous system (CNS) pathways are promising targets for the development of the most recent weight-loss therapies. Moreover, while starting an anti-obesity medical treatment, the clinicians should pay particular attention to possible concomitant obesogenic prescription medications, including all drugs in the classes of glucocorticoids, β-blockers, antihistamines, as well as selected agents in the classes of antidepressants, antipsychotics, antidiabetics, and contraceptives that are progestin-only [18]. Based on the analysis of national United States (US) databases, it has been observed that a quarter of the American population is assuming at least one of these drugs which are significantly associated with worse weight-loss outcomes [19]. For this reason, relevant scientific data are stressing the message that clinicians, tackling obesity, should try to minimize the use of obesogenic drugs and focus on prescribing agents that are weight neutral or that trigger weight loss, when those options are available and appropriate [19].

Another innovative approach aimed to maximize weight loss is represented by the use of targeted poly-pharmacology or unimolecular poly-agonists displaying activity upon multiple receptors. These include Melanocortin-4 receptor (MC4R) agonist/Glucagon-like peptide-1 receptor (GLP-1R) agonist combination [20–22].

In severe cases of obesity, bariatric surgery may be a viable option that can produce profound weight loss and may lead to diabetes and dyslipidemia remission, regardless of the procedure type [23]. However, it should be disclosed that surgery is also associated with an increased risk of developing obesity-related comorbidities and possible weight recovery in subsequent years [24, 25].

Although the fields of obesity research and related drug discovery have seen many exciting developments, only a few investigational agents are likely to meet the required criteria and to advance into the marketplace [26]. The related shortage of useful and authorized treatments leaves an open field to the improper use of drugs or dietary supplements whose safety and efficacy have not been confirmed. The use of various weight-loss supplements, such as dietary supplements and herbal products, is gaining worldwide acceptance, but qualitative and quantitative monitoring of pharmaceutical agents present in weight-loss supplements are needed [27].

In the present review we i) discuss the criteria required for a valuable anti-obesity drug; ii) summarize the risk and rewards of the most common anti-obesity medicines, and; iii) assess the potentiality and risks of pseudoephedrine in obese patients.

## The ideal anti-obesity drug

Given the multifactorial pathogenesis of obesity [1], its treatment involves an integrated approach between different intervention modalities. The first and fundamental therapeutic approach to curb the pandemic problem of obesity must be a change in the lifestyle through an adequate diet and the practice of a regular physical activity program adapted to individual abilities and state of health [28]. The pharmacological treatment should take place only after a poor effectiveness of diet and exercise in either inducing or maintaining weight-loss has been demonstrated. Currently not many pharmacological options are available and some of the drugs offer limited advantages over lifestyle intervention, and also the cost and side effects require that their use should be restricted to particular cases [29]. The properties of an ideal anti-obesity drug would be to produce a sustained decrease in body fat and/or visceral fat in a dose-dependent manner [30]. More specifically, the drug would have to decrease appetite, be active in the long-term, and preferably not producing tolerance or rebound effects. The definition of the benefit of an ideal anti-obesity treatment should not be restricted to the evaluation of the amount of body weight lost during the treatment but rather extended to the improvement of several comorbid conditions related to obesity [31, 32]. Furthermore, it should be inexpensive and easy to use because obesity is a condition that overtly affects individuals belonging to a low socioeconomic status, whereby affordability and availability become two decisive factors [33]. In addition, the ideal drug should have a simple regimen of administration (oral or weekly) in order to facilitate patient adherence [32]. Another ideal characteristic should be that the drug acts pleiotropically, improving other clinical aspects such as control of blood pressure or lipids, quite often associated with obesity [34].

However, the utmost complexity of the neurobiology of this disorder, with its redundant pathways, reduces the ability to discover a single-acting drug, suggesting that multiple approaches with different mechanisms are needed to produce a substantial and persistent weight-loss.

## Anti-obesity medications (AOMs): a large panel of treatment options

**Fig. 1:**
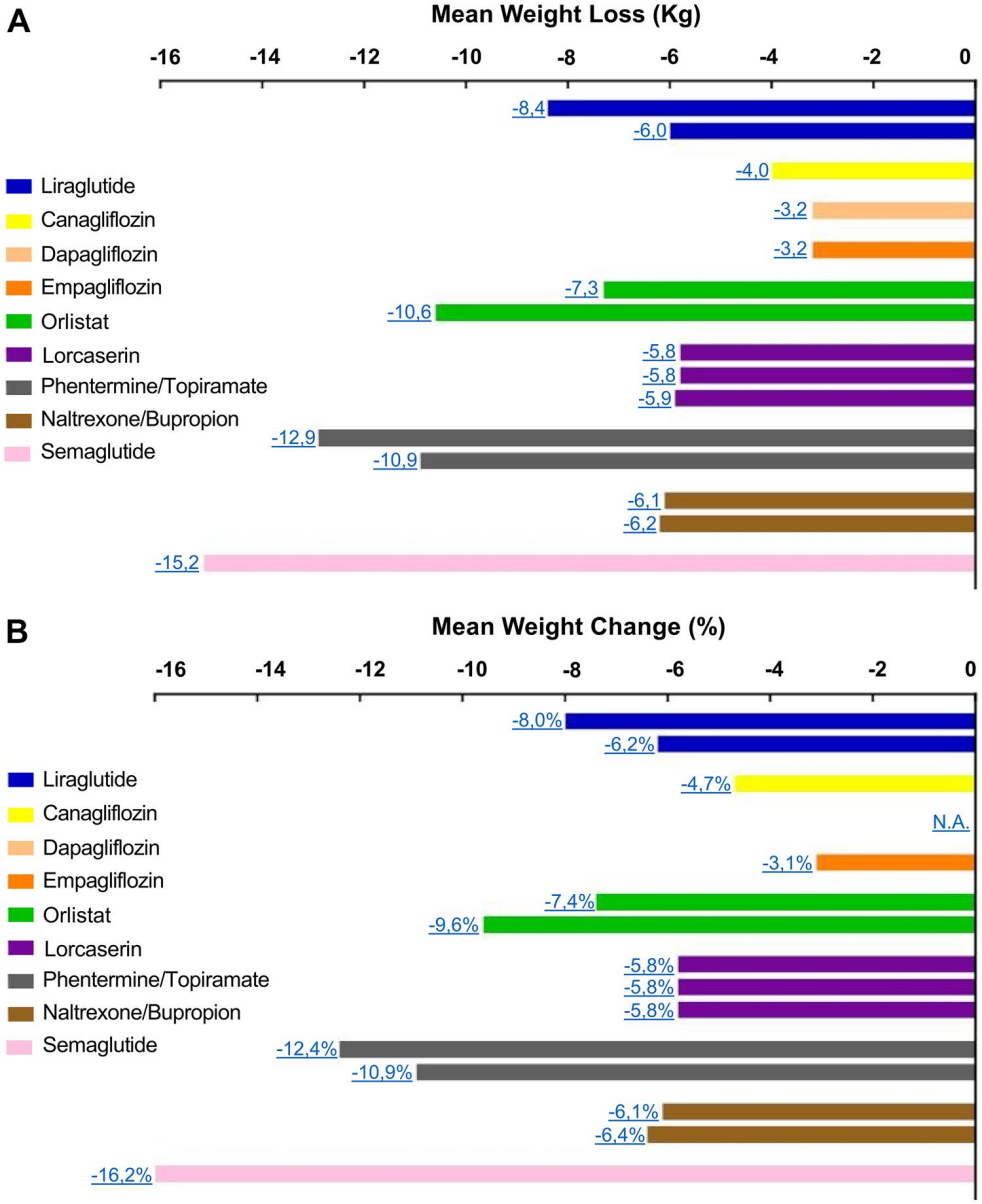
Effect of available anti-obesity drugs: Mean weight loss expressed in Kg (**A**) and mean weight change expressed in % (**B**) at the last-observation-carried-forward (LOCF), reported in yearly trials with different drugs at the highest dose (N.A. not-available data)

Despite the wide variety of molecules available, to date obesity does not yet have a definitive drug therapy. All anorectic drugs act through the most varied mechanisms of action, favoring a reduction in food intake and in the absorption of nutrients [35]. These include GLP-1R agonists [36–38], Type 2 sodium-glucose cotransporter (SGLT2) inhibitors (which, although associated with weight loss in people with type 2 diabetes due to their mechanism of action, are not generally considered anti-obesity drugs) [39–41], sympathomimetics [42, 43], serotoninergic system drugs [44–46], opioid µ receptor antagonists/weak inhibitor of neuronal dopamine and norepinephrine reuptake inhibitors [47, 48], and pancreatic lipase inhibitors [49, 50] (Fig. 1). In particular, all these medications are currently approved by both the Food and Drug Administration (FDA) and the European Medicines Agency (EMA), with the exception of sympathomimetics and serotoninergic drugs, which have been approved, so far, only by FDA. In front of such a wide range of options, as well as of the unmet need for every obese patient, the task to accomplish remains finding out the optimal individually tailored therapeutic regimen to treat obesity.

## Ephedra compounds

**Fig. 2:**
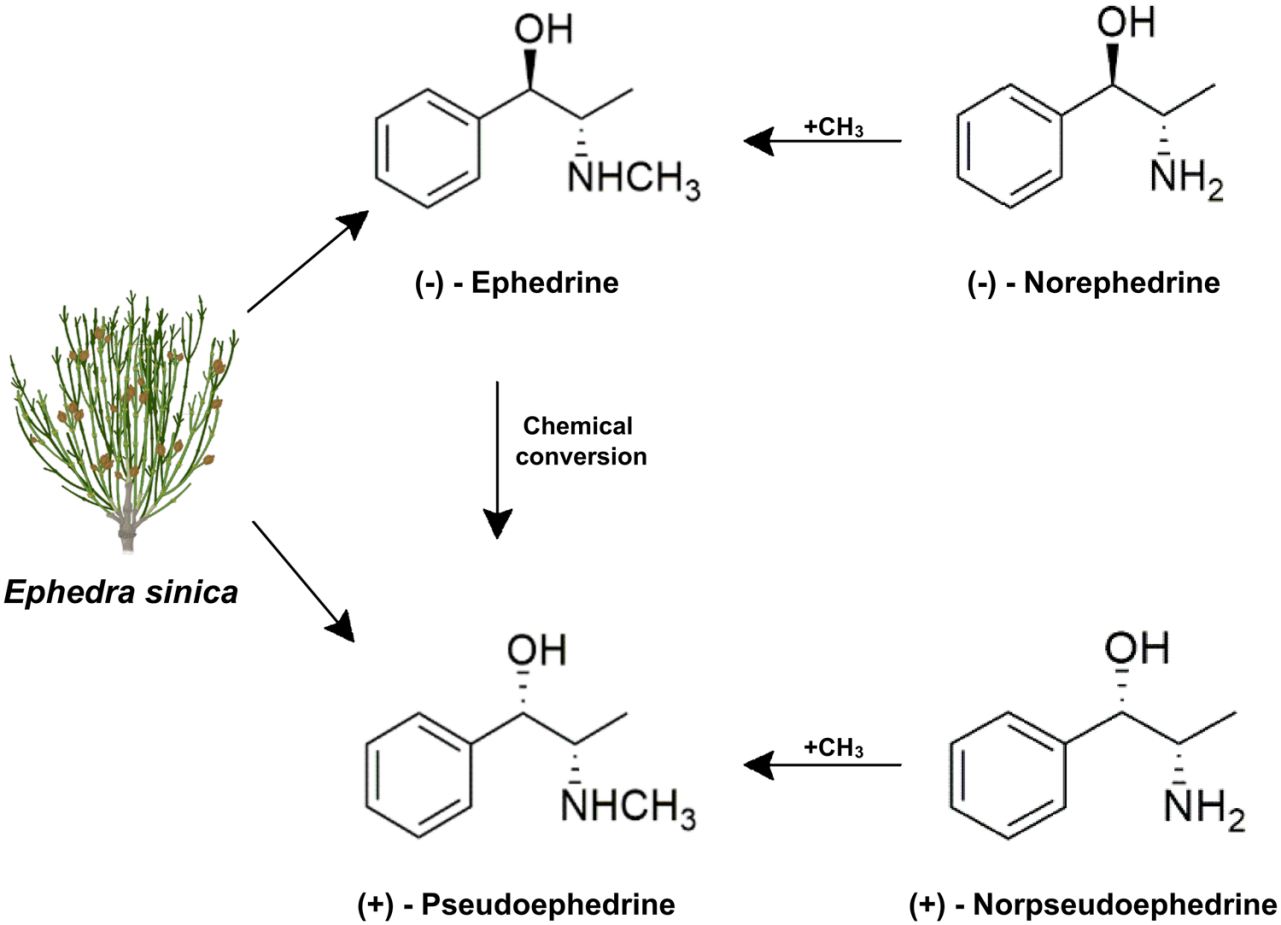
Structure of alkaloids from *Ephedra sinica*: *Ephedra sinica* was the first species of *Ephedra* used therapeutically in China. E. *sinica* has a strong pine odor and astringent taste, which accounts for its Chinese name (Ma-huang) which can be translated as ‘yellow astringent’. The wide range of pharmacological activities showed by this plant are related to the content of ephedrine-type alkaloids. (-)- Ephedrine and ( +)-Pseudoephedrine occurs as the main sympathomimetic alkaloids

One of the oldest medicinal herbs is probably *Ephedra*, also known as “Ma-huang”, which presents a long history in traditional Chinese medicine as a treatment for bronchial asthma, colds, headache, and nasal congestion [51]. In particular, *Ephedra sinica*, a member of the *Ephedraceae* family, is the most commonly used in preparations and extracts. *Ephedra* includes several sympathomimetic substances, such as ephedrine, pseudoephedrine, nor-ephedrine, methyl-ephedrine, and methyl-pseudoephedrine, all substances with molecular structures related to catecholamines and amphetamines [52]. The alkaloid content of *Ephedra* species is highly variable, depending upon the type, the parts of the plant used and the method of extraction, the most represented being ephedrine and its stereoisomer pseudoephedrine (Fig. 2). The latter substance is characterized by an ephedrine-like effect but less pronounced cardiac action, less adverse effects and higher diuretic activity [53, 54]. Despite its long history, the use of *Ephedra* herb has declined throughout the years, due to the lack of concrete effectiveness and the poor labeling for possible toxicity. However, recently, many companies started marketing dietary supplements for weight reduction, containing *Ephedra* extract. The wide availability of these non-prescription products and the wrong concept of safety have increased the misuse and abuse of the herb and relative mounting evidence of possible hazards.

### Pharmacodynamics and pharmacokinetics

**Fig. 3:**
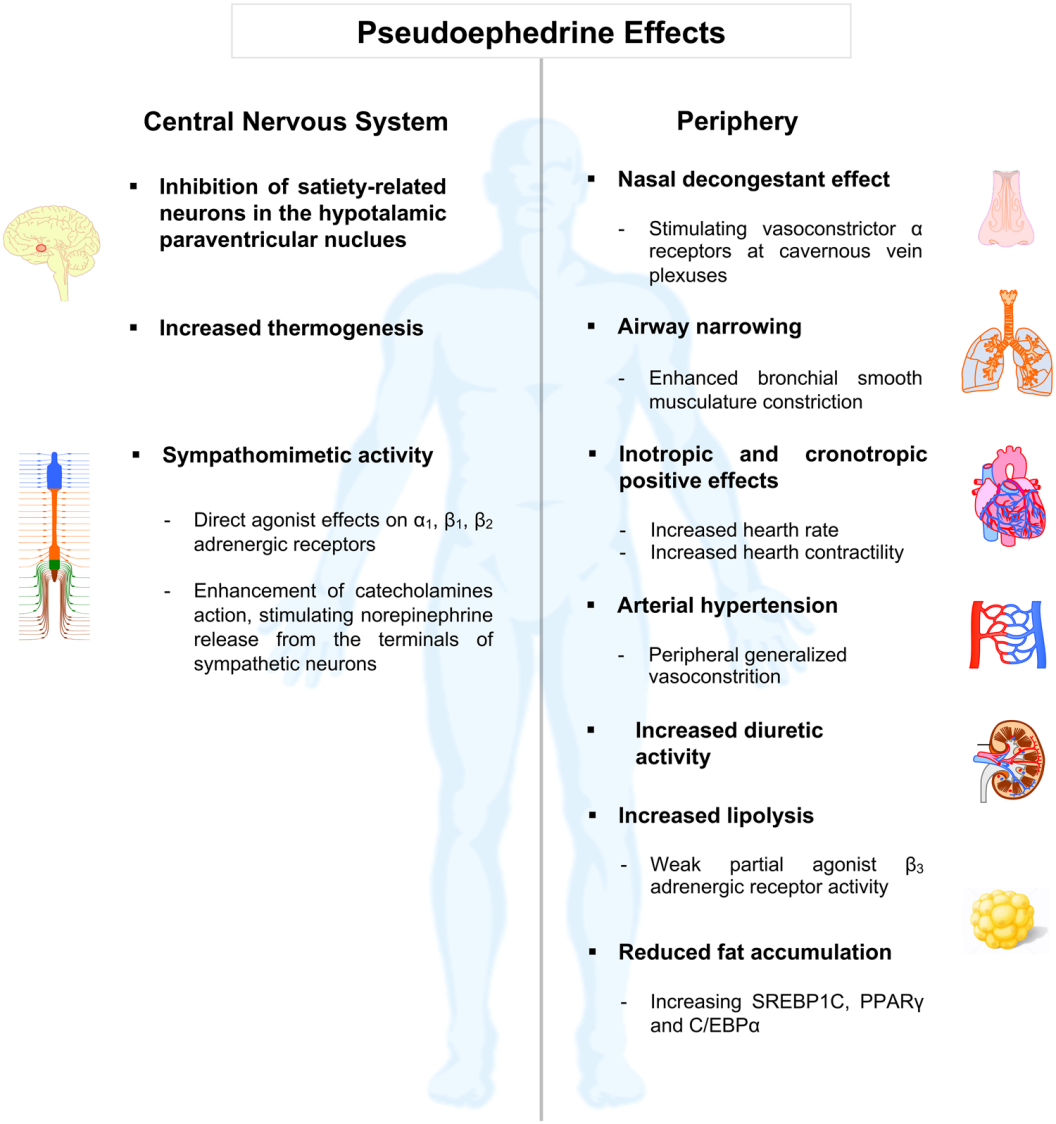
Effects of Pseudoephedrine: Pseudoephedrine is a sympathomimetic agonist that also displays indirect sympathetic activation enhancing the release of norepinephrine from sympathetic neurons. This pharmacological mechanism seems to account for most of the pseudoephedrine’s therapeutic efficacy, as well as its notable adverse effects. Characteristic effects of adrenergic receptor stimulation include enhanced cardiac rate and contractility, peripheral vasoconstriction, bronchodilation, and central nervous system (CNS) stimulation. The vasoconstrictor, mainly, and bronchodilator effects explain the traditional use of pseudoephedrine as a nasal decongestant and anti-asthmatic. CNS stimulation encompasses the inhibiting effect on satiety-related hypothalamic neurons that, combined with the thermogenic effect and the increased lipolytic activity, is purported to afford the renowned slimming effect

From a pharmacodynamics perspective, pseudoephedrine presents a sympathomimetic action both directly, by exerting agonist activity on β_1_, β_2_ and α_1_ adrenergic receptors, and indirectly, by inducing the release of norepinephrine from sympathetic neuron terminals, enhancing the effects of catecholamines [55]. Ephedrine and pseudoephedrine additional action of depleting the endogenous catecholaminergic reserves may explain the onset of tachyphylaxis after repeated dosing [56]. By virtue of their molecular structure, ephedrine and pseudoephedrine stimulate α adrenergic receptors at cavernous vein plexuses, determining its nasal decongestant effect [57]. Such apparently basic pharmacological mechanism accounts for either its therapeutic, as well as for the most evident adverse effects [58]. Pseudoephedrine increases hearth rate and contractility, induces constriction of bronchial and peripheral vessels smooth muscle, and affects the function of CNS [59] (Fig. 3). Because of these pharmacodynamic characteristics, patients under treatment or who recently discontinued therapy with monoaminoxidase inhibitors (MAOi), should not take pseudoephedrine for the increased risk of hypertensive episodes, such as paroxystic hypertension and malignant hyperthermia. Moreover, pseudoephedrine enhances the effects of other sympathomimetic drugs, thus increasing the risk of intense vasoconstriction and consequent possible hypertensive seizures; similarly, it is not recommended its use concomitantly with reversible inhibitors of monoaminoxidase A (RIMA) and ergot alkaloids, for the increased risk of vasoconstriction and/or hypertensive crises and severe arrhythmias [60, 61]. As a sympathomimetic amine and precursor of amphetamine-like metabolites, pseudoephedrine owes its slimming properties to its anorectic action exerted through the inhibition of the activity of hypothalamic neurons of satiety, located in the hypothalamic paraventricular nucleus (PVN) and distinctively involved in the regulation of food intake, energy and sleep [62]. Furthermore, Vansal and Ferrel have proven that ephedrine isomers are able to interact with β3 adrenergic receptors involved in lipolysis and thermogenesis [56]. Recent studies have shown that both ephedrine and pseudoephedrine are able to reduce fat accumulation by increasing the levels of down-regulators of the lipogenic transcriptional factors, such as sterol regulatory element-binding protein 1C (SREBP1C), peroxisome proliferator-activated receptor gamma (PPARγ), and CCAAT/enhancer-binding protein α (C/EBPα) [63, 64]. The previously mentioned CNS stimulant activity, supported by its ability to cross the blood–brain barrier, combined with the thermogenic action, the anti-lipogenic activity, and the appetite suppressant effect [65], led several manufacturers to include pseudoephedrine and *Ephedra* compounds in the formulation of diet supplements to promote enhanced weight loss in obesity and improved performance in endurance training or body-building [66].

Pseudoephedrine does not undergo hepatic first-pass metabolism and its gastrointestinal absorption is rapid and complete. The peak of plasma concentration is 500–900 µg/l and is reached about 2 h after oral administration of 180 mg pseudoephedrine. The plasma half-life is about 5–8 h, but the plasma concentrations vary considerably between individuals [67, 68]. Pseudoephedrine is resistant to the action of monoaminoxidase (MAO) and is excreted, mainly in an unmodified form, through the renal emunctories. Several pharmacokinetic studies have shown that at high urine pH, pseudoephedrine, as a weak base, is non-ionized, thus it is easily reabsorbed from the renal tubules, whereas at low urine pH, ephedrine is electrically charged and is cleared faster [69, 70]. Only a 1% fraction of pseudoephedrine is eliminated via the liver, by N-demethylation and formation of nor-pseudoephedrine (catine). Additionally, it has been demonstrated that there was no correlation between the half-life of pseudoephedrine and the severity or the amount of symptoms experienced by the subjects [71].

### Clinical data

After the first studies in the 1970s, the use of *Ephedrine* products was widespread in the following decades in Europe and in North America. Such exceeding consumption was promoted by the classification of these substances as nutritional supplements for slimming [72]. Despite a comprehensive characterization of the mechanisms of action of pseudoephedrine, clinical data on the use of this compound in obesity are quite surprisingly limited. Only a single placebo-controlled weight-loss study of a slow-release formulation of pseudoephedrine (120 mg/day), conducted in 72 patients for 12 weeks, is available in the literature [73]. The two groups in the study had similar anthropometric characteristics (baseline BMI 29.2 kg/m^2^ in the pseudoephedrine treatment group vs 28.5 in the placebo group). Weight loss at the end of the study overlapped in the two groups (4.6 kg pseudoephedrine vs. 4.5 kg placebo), with no statistical significance at any intermediate point of the study. Also, there was no difference in appetite reported by patients in the two subgroups. Controlled clinical studies aimed at verifying the effects of higher doses of pseudoephedrine are not available. Furthermore, there are no evidence that pseudoephedrine would cause less dependence than ephedrine [73].

However, several studies have been conducted to investigate the association between the consumption of food supplements and drugs containing ephedra compounds and the onset of adverse events [74]. Between 1997 and 1999, the FDA received more than 140 reports of adverse events associated with the use of dietary supplements containing ephedra alkaloids. Among the 87 events that have been definitively, probably, or possibly related to the use of these food supplements, 10 resulted in death, 13 in permanent damage, while the remaining cases outcome to a full recovery. The most frequently observed events included hypertension, palpitations and, within neurological symptoms, seizures and stroke [75]. Using the comprehensive database Adverse Reaction Monitoring System of the FDA, Samunek and colleagues assessed the possible cardiovascular toxicity associated with the use of dietary supplements containing *Ephedra*. The authors have assessed a time correlation between ephedra consumption and 37 cases of stroke, half of which hemorrhagic, 10 cases of myocardial infarction and 11 cases of sudden death. They also concluded that, although pathogenesis is not fully defined yet, the cardiovascular toxic effects associated with ephedra were not limited to massive doses and may be associated with serious complications even in the apparent absence of underlying cardiovascular disease [76]. Analyzing a stroke registry since 1988, Cantu et al. found that 22 out of 2500 stroke patients manifested the event in a way associated with taking an over-the-counter (OTC) cough and cold sympathomimetic drug, containing phenylpropanolamine and pseudoephedrine. The relationship with the drug was established on the basis of a clear temporal association and after excluding other plausible known causes. Almost all events were found to be hemorrhagic and the tests carried out support the hypothesis that a hypertensive crisis and/or a similar vascular mechanism may lay at the basis of the event [77].

An extended meta-analysis, assessing the safety of *Ephedra* and ephedrine containing products for weight loss and athletic performance, reviewed the results of 50 controlled trials, all case reports for *Ephedra* compounds in the FDA MedWatch, as well as all case reports identified in published literature and a very large file of symptoms reported to a manufacturer of *Ephedra*-containing dietary supplements. The authors collected sufficient evidence to conclude that the use of ephedrine and *Ephedra* compounds resulted in two-to-three times increased risk of psychiatric symptoms, autonomic symptoms, upper gastrointestinal symptoms, tachycardia and hypertension compared to placebo. They also found a high number of case reports of serious adverse events occurring, in the absence of other possible causes, in young patients who used *Ephedra* or ephedrine, strengthening the possibility of a causal relationship [78]. Consequently, it has become difficult for manufacturing companies to oppose to the withdrawal of these combinations by the FDA, that, finally, in 2004 banned *Ephedra* and ephedrine products due to the unpredictable risk of adverse events. [79].

Despite the severity of the measures taken, the use of *Ephedra* alkaloids has not been stopped, supported also by the availability of these substances on the Internet and by the wrong, populist belief that, as they are of natural origin, these products are characterized by an excellent safety profile. As proof of this, numerous published reports underline such unpredictability of the effects of Ephedra alkaloids, and how numerous and variable may the related adverse events be [57]. In particular, the adverse events of pseudoephedrine are mainly concerning the cardiovascular system, supported by the increased availability of catecholamines and by the subsequent overstimulation of the adrenergic receptors. In addition to hypertension [80], regarded to as the most common adverse event, numerous cases of angina pectoris and myocardial infarctions have been reported also in young, healthy patients with no risk factors and after assumption of the recommended dose of an OTC cold remedy containing pseudoephedrine [81–84]. In many of the reported cases, the absence of a significant coronary disease and the other tests performed were consistent with an acute myocardial infarction caused by acute vasospasm caused by the adrenomimetic effects of the medication [85]. Of particular interest is the report by Fidan and colleagues, who described a case of ST-elevation myocardial infarction (STEMI) after the use of pseudoephedrine. This is the first study in literature in which the cardiac toxic effects of pseudoephedrine were confirmed by measuring the serum drug concentration. A causal relationship between drug intake and the cardiac event has then been established [86].

An identical pathophysiological mechanism was postulated to justify the onset of ischemic colitis following oral administration of pseudoephedrine-based decongestants [87]. This adverse event occurred in the absence of a major cardiovascular risk factor, hemodynamic instability, or hypercoagulability state, thus excluding major conditions predisposing the onset of this acute event. Given the clearcut temporal relationship between ingestion of the drug and the occurrence of symptoms, the OTC medication was, therefore, the most likely cause of this ischemic colitis [87]. In other case reports, ischemic colitis occurred with variable dosage and duration of treatment with pseudoephedrine, ranging from 60 to 900 mg per day and for a period between 5 days and 2 years [88–91]. Together, these data underscore the need to collect a scrupulous drug history, including the ingestion of OTC and herbal supplements in ischemic colitis patients, with special regard to young patients.

The effects of pseudoephedrine are not exclusive to the cardiovascular system. The mixed-sympathomimetic properties of the ephedra compounds also influence the central nervous system, leading to increased presynaptic calcium-independent release of catecholamines, as well as to postsynaptic β1 and β2 activation [92]. This stimulating action on the CNS may become manifest during treatment through the onset of restlessness, insomnia and anxiety with consequent reduced concentration capacity and alteration of the mood state [93]. Therefore, such increase in noradrenergic tone, combined with dopamine release, can also lead to the onset of psychotic symptoms [94, 95]. This is not surprising when considering the chemical structure of these compounds, which can be classified as natural amphetamines [96]. The similarity with these substances does not end in the analogy of the chemical asset, but can be extended to the clinical phenomenology of induced psychoses, characterized mainly by a paranoid phenotype with delusions of persecution, and auditory and visual hallucinations [97] (Table 1). The first cases reported in the literature date back to the early 1970s and, since then, the number of case reports has steadily increased [92, 98–100]. Most ephedrine/pseudoephedrine-induced manic episodes involve a pre-existing mood disorder history, suggesting that ephedrine may exacerbate pre-existing mood disorders, or precipitate a previously unproven one [101–104]. Consistently with these data, several cases have been described in which the use of products containing pseudoephedrine has been correlated with the onset of manic and psychotic symptoms in a schizoaffective patient after a period of remission of 10 years [105]. Others have reported two mania episodes triggered by pseudoephedrine in the context of a bipolar manic disorder [106]. Nevertheless, there are also reports of ephedrine-induced mania in the absence of a previous history of depression or other mood disorders, suggesting that these drugs may potentially initiate sustained mood dysregulation [107]. Such association of behavioral side effects with the use/abuse of OTC cold medications containing ephedrine and pseudoephedrine, has also been shown in children [108, 109]. All three reported cases describe similar clinical pictures of severe acute psychosis, which required several days of hospitalization and a multidisciplinary evaluation [110, 111]. The authors conclude that intoxication by this class of compounds should be included in the differential diagnosis of new-onset psychosis.

**Table 1:** Recent reports of psychiatric disturbance following the use, misuse or abuse of pseudoephedrine

TITLE	TEXT	REFERENCE
Psychiatric symptoms associated with ephedra use	According to a recent *in vitro* study, the most important actions of ephedrine’s alkaloids are as substrates of the noradrenaline transporter, followed by substrate activity at the dopamine transporter. Thus, these alkaloids increase the brain’s release of noradrenaline and to a lesser extent, dopamine A 39-year-old female took a diet aid (tested by the manufacturer and said to contain 6 mg ephedra alkaloids per capsule, 12 mg per dose) at recommended doses. Her mother reported that the daughter experienced insomnia, hallucinations, psychosis and delusions one year after product initiation She required hospitalization in a psychiatric facility for 40 days, with ongoing problems, including terror, panic and forgetfulness	[129]
Mania following the use of a decongestant	…a 56-year-old woman with no psychiatric history who had a manic episode after taking a decongestant containing pseudoephedrine	[130]
Herbal Drugs of Abuse: An Emerging Problem	Ma-huang, containing the dried stems of *Ephedra equisetina*, contains the psychoactive alkaloids ephedrine, norephedrine, pseudoephedrine, and norpseudoephedrine. Ephedrine alkaloids are structurally related to amphetamines and act as direct-acting sympathomimetics with nonspecific α- and β-adrenergic agonist activity	[131]
Clinical characteristics of cough mixture abusers referred to three Substance abuse clinics in Hong Kong: a retrospective study	Psychotic disorders were the most common psychiatric diagnosis in cough mixture–dependent patients attending 3 substance abuse clinics in Hong Kong. Common ingredients in local cough mixture were promethazine, ephedrine, pseudoephedrine, codeine, and hydrocodone, while dextromethorphan was infrequently detected. Co-dependence of cough mixture with zopiclone or zolpidem was often found	[132]
Symptoms of major depression after pseudoephedrine withdrawal: a case report	Pseudoephedrine is an effective and commonly used congestion remedy known to have stimulant properties. Thus, it is surprising how few reports describe its interaction with mood. To our knowledge, this is the first report of simple unipolar depression appearing to benefit from initiating pseudoephedrine, and then significantly worsening during the withdrawal period	[133]
Neuroleptic malignant syndrome in an adolescent with CYP2D6 deficiency	In September 2009, a 16-year-old male (weight: 65.5 kg, height: 1.83 m, BMI = 19.6 kg/m2) was admitted to a pediatric neurology unit due to dystonia. A month before, the patient became withdrawn and developed insomnia and twisting movements of the left extremities. These symptoms began after a 1-month of using medication containing dextromethorphan and pseudoephedrine for recreational purpose	[134]
Benefits, limits and danger of ephedrine and pseudoephedrine as nasal decongestants	It further seems that the severe adverse cardiovascular and neurological effects reported with these amines, of unpredictable onset and potentially associated with low doses in the absence of any relevant history, should lead ENT physicians not to resort to them to treat common cold and to exercise the greatest rigor in assessing the cost/benefit trade-off in prescribing them for allergic rhinitis. Given these risks, distribution should be regulated, and over-the-counter sales should be avoided The study reported 22 episodes of arterial hypertension, 15 of convulsion and 4 cases of stroke after oral intake of medication containing pseudoephedrine	[57]
Misuse of OTC drugs in Poland	Misuse of OTC medications became common especially among young people and the recreational use of the substances, which may cause inability to concentrate, hallucinations, dizziness, seizures, hyperexcitability and/or even psychosis, has significantly increased recently	[135]
A case report of patient who had two manic episodes with psychotic features induced by nasal decongestant	Phenylephrine, pseudoephedrine and ephedrine are the sympathomimetic drugs that have been used most commonly in oral preparations for the relief of nasal congestion. These drugs stimulate the central nervous system that is affected by the α and β adrenergic agonism. Sympathomimetic agents used in the treatment of flu and common cold with ephedrine and pseudoephedrine are case reports. That the manic and psychotic episodes are triggered. In this article, we would like to present a bipolar manic disorder with two manic episodes and both of them triggered by influenza drugs	[106]
Can nasal decongestants trigger a manic episode?	Manic episodes with/without psychotic properties, psychotic attacks, and chronic psychosis triggered by ephedrine and pseudoephedrine have been reported in the literature …a 13-year-old girl developed manic symptoms after receiving an amount 6 times greater than the recommended 60 mg dose of pseudoephedrine prescribed for nasal congestion and started treatment for drug-induced affective disorder	[136]

More recently, simultaneous bilateral acute angle-closure crisis (AACC), a sight-threatening ocular emergency, triggered by cold and flu, as well as by preparations containing compounds with sympathomimetic properties [112, 113], or by a single oral dose of pseudoephedrine were documented [114]. The authors point out that the symptoms of simultaneous bilateral AACC may overlap with the flu-like symptoms for which the medications potentially triggering AACC are taken. Although the simultaneous bilateral onset of visual disturbance leads to a clinical suspicion of a central neurological pathology, the progressive nature of symptoms and their onset following the intake of a suspect drug should prompt Clinicians to consider drug-induced simultaneous bilateral AACC as a possible diagnosis [115]. In such cases, an urgent ophthalmological assessment is required.

Adverse drug reactions due to pseudoephedrine not only include numerous cases of pigmented [116, 117] and non-pigmented skin eruptions [118–120], but also some generalized scarlatiniform [121] or eczematous eruptions [122–124]. Moreover, cases of recurrent acute generalized exanthematous pustulosis and severe mucosal involvement have been described [125]. Mayo-Pampín E. et al., reported a case of acute generalized exanthematous pustulosis (AGEP), a severe and rare skin disease generally induced by certain antibiotics such as aminopenicillins and macrolides [126]. In all these cases, the causal role of pseudoephedrine has been confirmed by patch tests that provide the diagnosis of T lymphocyte-mediated hypersensitivity caused by this pharmacological compound [127]. Overall, the cases described suggest that, if such hypersensitivity is suspected, it is crucial for an appropriate diagnostic approach.

## Conclusions

When compared to other pharmacological options for the treatment of obesity, the above evidence suggests that pseudoephedrine is absolutely contraindicated, in addition to pregnancy and breastfeeding, in all pre-existing cardiovascular and neuropsychiatric diseases. At any rate, risks arising from the use of pseudoephedrine depend significantly upon individual susceptibility, which, at the present state of knowledge, is not known, and, therefore, scarcely predictable, for all compounds of this class [75]. It should also be kept in mind that, even in clinical situations in which it is mandatory to achieve rapid weight loss (e.g., cases of severe obesity in which major surgery, including bariatric surgery, is indicated) the patient very often is already affected by cardiovascular comorbidities [128], representing, per se, a specific contraindication to the use of pseudoephedrine or congeners. Even in the absence of specific cardiovascular comorbidities, one should consider that in patients suffering from severe obesity, the use of pseudoephedrine can lead to unpredictable development of frank pathological conditions. A further aspect to consider is the neuro-psychological one, with special regard to the variable degree of individual susceptibility to pseudoephedrine. The unstable psychological structure of an obese individual, oppressed by the goal of loosing weight "at all costs", entails, given the intake of these substances with addictive properties, an increased risk of breaking the psychological balance, which can hesitate in behavioral disorders that are not always reversible [105].

Given the scanty clinical literature data, absolutely insufficient to draw reliable conclusions about its efficacy, the risk/benefit profile of pseudoephedrine in obesity is strongly leaning in favor of an increased and unpredictable cardiovascular and neuropsychiatric risk. In addition, a relevant impact on the toxic potential of pseudoephedrine in obesity is certainly represented by the number of OTC products, which, not needing a prescription, become the reason of uncontrolled occurrence of non reported, serious adverse reactions. Consequently, the treatment of obesity based on self-medications has became a quite challenging issue.

The frank and/or latent comorbidities, which characterize a chronic pathology such as obesity, expose these patients to a high risk of developing either severe arrhythmic events, and/or non-reversible neuropsychiatric disorders. The toxicological aspect linked to possible tachyphylaxis, which requires patients to move rapidly to higher doses to achieve the same effect over time, is also not negligible. In light of the above considerations, in light of the unfavorable risk/benefit ratio presented by pseudoephedrine associated with its addictive potential, there appear no valid reasons for its systematic use in obesity of any order and degree.
